# miRNAs expression of oral squamous cell carcinoma patients: Validation of two putative biomarkers

**DOI:** 10.1097/MD.0000000000014922

**Published:** 2019-03-15

**Authors:** Cintia Micaela Chamorro Petronacci, Mario Pérez-Sayáns, María Elena Padín Iruegas, José M. Suárez Peñaranda, Alejandro Ismael Lorenzo Pouso, Andrés Blanco Carrión, Abel García García

**Affiliations:** a Oral Medicine, Oral Surgery and Implantology Unit, Faculty of Medicine and Dentistry, Santiago de Compostela University, Instituto de Investigación Sanitaria de Santiago (IDIS), Santiago de Compostela; b Human Anatomy and Embryology Area, Faculty of Physiotherapy, Department of Functional Biology and Health Sciences, Pontevedra, Vigo University; c Pathological Anatomy Service, Hospital Clinico Universitario de Santiago (CHUS), Santiago de Compostela, Spain.

**Keywords:** expression profile, microRNA, oral neoplasm

## Abstract

microRNA expression patterns have provided new directions in the search of biomarkers with prognostic value and even in the search of novel therapeutic targets for several neoplasms. Specifically, miRNAs profiling in oral squamous cell carcinoma (OSCC) represents a web of intrigue in the study of oral carcinogenesis. The objective of the present study was twofold:

1. to analyze miRNA profiling and estimate related biological pathways and,
2. to assess miRNAs profiles prognostic values in a cohort of OSCC-affected patients.

The first study phase comprised case-control groups: A) 8 OSCC-affected patients and 8 healthy controls. Microarray technology (Affymetrix miRNA Array Plate 4.1) was used for miRNAs expression profile. Deregulated miRNAs were studied using Diana Tools miRPath 3.0 to associate miRNA targets with molecular pathways via Kyoto Encyclopedia of Genes and Genomes (KEGG). In a second phase, 2 miRNAs chosen for the subsequent RT-qPCR validation were studied in a second OSSC cohort (n = 8).

Microarray analysis identified 80 deregulated miRNAs (35 over-expressed and 45 under-expressed). Two miRNAs (miR-497-5p and miR-4417) were chosen for further validation via RT-qPCR. Prognostic analysis did not ascertain relevant relation between miR-497-5p or miR-4417 expression and clinical or pathological parameters, except high miR-4417 in the case of nodular affectation (*P* = .035) and diminished miR-497-5p radiotherapy-treated patients (*P* = .05). KEGG analysis revealed that deregulated miRNAs were implicated in several biological pathways such as Proteoglycans in cancer.

Our data suggest an altered miRNAs profiling in OSCC-affected patients. We have verified the altered expression of miR-497-5p and miR-4417 in OSCC samples and related the deregulated miRNAs with the ‘proteoglycans in cancer’ pathway. Further longitudinal studies with large samples are warranted to confirm the present findings.

## 1 Introduction

Oral squamous cell carcinoma (OSCC) is one of the most frequent cancers worldwide. The 5-year survival rate of OSCC patients is only about 50% because most patients are diagnosed in advanced cancer stages.^[1]^ Despite biological and technological advances, its prognosis has not improved in the last few decades, and its incidence has increased. Owing to the severity and complexity of OSCC, improvements in diagnostic and prognostic tools in the clinical setting are needed. The determination of affected molecular pathways permits the identification of biomarkers that might be used to make an early diagnosis or anticipate tumor progression.

microRNAs (miRNAs) are small, noncoding RNAs (18–25 nucleotides long) that regulate protein expression at the post-transcriptional level. miRNAs participate in different physiological processes such as cell development, proliferation, differentiation, apoptosis, immune response and angiogenesis.^[2,3]^ The deregulation of specific miRNAs can lead to cancer initiation and progression.^[4]^

Emerging barriers limit the clinical translation of this marker to the study of head and neck cancers due to inconsistent miRNA profiles.^[5]^ Current literature relate these discrepancies with different matters such as the type of sample (frozen tissue, formalin-fixed paraffin-embedded cell lines), localization and origin (oral cavity, pharynges, or larynges),^[6]^ platforms used, and lesion type analyzed (from premalignant lesions to advanced lesions with metastasis).^[7,8]^

miRNA expression profiles studies demonstrate differences among populations.^[9]^ The majority of the miRNA profiling reports on OSCC have been based on Asian samples. Given that an epigenetic mechanism underlies the origin of many cancers, several authors agree on the necessity of studying miRNA profiles from different populations to improve the quality of evidence.^[10]^ Also, the ongoing discovery of new miRNAs forces investigators to develop new studies with updated technologies to identify such miRNAs.

Hence, the objective of the present study was twofold:

1. to analyze miRNA profiling and estimate related biological pathways and,
2. to asses miRNAs profiles prognostic values in a cohort of OSCC-affected patients.

## 2 Materials and methods

### 2.1 Patients and samples

All procedures were carried out with the adequate understanding and written consent of the subjects in accordance with the *Declaration of Helsinki*. The approval of the Clinical Research Ethics Committee of Galicia Ref. 2015/132 (CEIC), currently known as the Comité Autonómica da Ética da Investigación (CAEI), was obtained for this study.

To determine the miRNA expression profile, a case-control study design was performed. OSCC samples were obtained from 16 patients treated by the Maxillofacial Surgery Unit of Complejo Hospitalario de Santiago de Compostela (Santiago Teaching Hospital) from 1994 to 2015. All samples were preserved at −80°C at the hospital's biobank (SERGAS). The keratinized gingiva surrounding a third molar, which was extracted, was extracted as a control sample in 8 healthy patients by the same unit in 2015.

### 2.2 Criteria and data

Inclusion criteria OSCC patients: all patients treated by Maxillofacial Surgery Unit with no history of cancer, radiotherapy or chemotherapy, adults (older than 18 years old), with access to their clinical data.

Exclusion criteria OSCC patients: patients younger than 18 years old, with previous history of cancer, radiotherapy and/or chemotherapy.

Inclusion criteria of healthy patients: adults, with the necessity of third molar extraction.

Exclusion criteria of healthy patients: previous history of cancer, radiotherapy and/or chemotherapy.

### 2.3 Bias

Previous history of cancer was determined as an exclusion to criteria to avoid molecular expression patterns from other possible primary tumors.

### 2.4 Study size

OSCC is a rare cancer with a prevalence of 3.5 cases/100.000 inhabitants in Europe. Study size was as huge as it was possible to obtain.

### 2.5 Variables

Clinical parameters noted from all patients were as follows: sex, age smoking (smoker, non-smoker, ex-smoker). Additionally, from OSCC patients following parameters were also noted: tumor localization, differentiation, stage, TNM classification, posterior necessity of radiotherapy and/or chemotherapy, relapse and vital situation (death or not).

### 2.6 RNA isolation

Total RNA was extracted using the mirVana miRNA Isolation kit as previously described.^[11]^ Sample RNA concentrations were measured with a NanoDrop 2000 system (Thermo Fisher Scientific, Waltham, MA), and RNA integrity (RIN) was assessed using NanoChip 6000 (Agilent, Santa Clara, CA) and Bioanalyzer (Agilent). The concentration and RIN had to be at least 200 ng/μl and 7, respectively.

### 2.7 miRNA profiling

Once total RNA was extracted, samples were labeled with the Affymetrix FlashTag Biotin HSR RNA Labeling Kit. The Affymetrix miRNA 4.1 array plate was used for the microarray, since it contains enough probes to identify 2578 human miRNAs. The analysis of the differential expression was developed using the software Transcriptome Analysis Computer 4.0 by Affymetrix (free access).

### 2.8 Enrichment analysis

Enrichment analysis for molecular pathways was performed with miRNAs that were downregulated or upregulated in OSCC with a statistically significant difference (*P* < .01). Kyoto Encyclopedia of Gene and Genomes (KEGG) analysis was carried out through Diana Tools mirPath v3.0 (free access). Results were determined following the ‘pathway-union’ criteria; *P* values were calculated by Diana software.

### 2.9 miRNA quantitative RT-PCR

Targets of those miRNAs with higher significance level in microarray analysis were searched in Diana Tools miRPath, and four miRNAs with OSCC known targets (miR-497-5p, miR-617, miR-4417, and miR-6825-5p) were selected to corroborate the results with RT-qPCR. RNU6B was used as the endogenous control as previously described.^[12]^ Retrotranscription was performed with the TaqMan MicroRNA Reverse Transcription Kit (Applied Biosystems) with the following thermal cycling profile: 16°C for 30 minutes, 42°C for 30 minutes, 85°C for 5 minutes, and finally, 4°C.

TaqMan microRNA assay (Thermo Fisher) was used for RT-qPCR with samples from 16 patients and 4 control individuals, with the following thermal cycling conditions: 40 cycles of 95°C for 10 minutes, 95°C for 15 seconds, and 60°C for 1 minute, following the manufacturer's instructions. Each sample was spotted thrice. CT values were normalized, and miRNA expression levels were calculated using CT comparative level: C_tSAMPLE_ − C_t__MEANNORMALIZERS._

### 2.10 Statistical analysis

For the first analysis, a standard descriptive analysis was performed, which included mean, standard deviation, frequency, and percentage.

For array data analysis, the Transcriptome Analysis Console 4.0 (TAC) developed a univariate ANOVA analysis that selected genes with ±2-fold change and *P* values <.05. Diana Tools miRPath 3.0 used Fisher exact test to relate miRNA targets and their molecular pathways. For the RT-qPCR statistical analysis, we first normalized the data. Next, we performed Pearson chi-square test, to check statistical differences between cases and controls. The relationship between clinical and pathological parameters and the expression of deregulated miRNAs were calculated using non-parametric statistics with the Mann-Whitney *U* test and the parametric chi-square test, depending on the application conditions. Kaplan-Meier analysis and the log-rank test were performed to identify survival differences in OSCC patients.

To verify the correlation of the expression between both miRNAs in the 2 different platforms, we developed a non-parametric correlation analysis by using Spearman rho, considering a good association value above 0.6. Differences were considered statistically significant when *P* < .05.

## 3 Results

### 3.1 Descriptive analysis

A total of 24 patients participated in this study (16 cases and 8 controls); the clinical and demographic parameters are summarized in Table 1. The TNM staging system could not be obtained in 1 case.

**Table 1:**
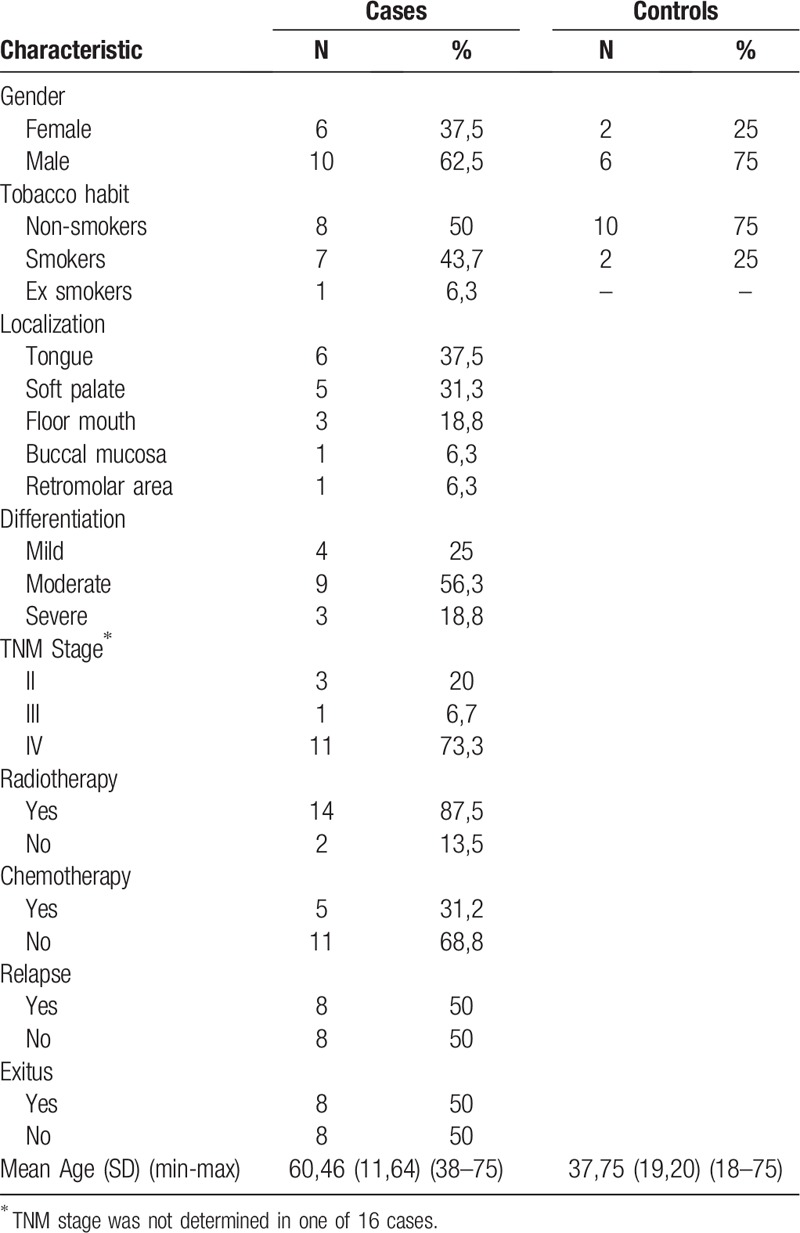
Descriptive analysis.

### 3.2 Profiling of differentially expressed miRNAs

**Figure 1:**
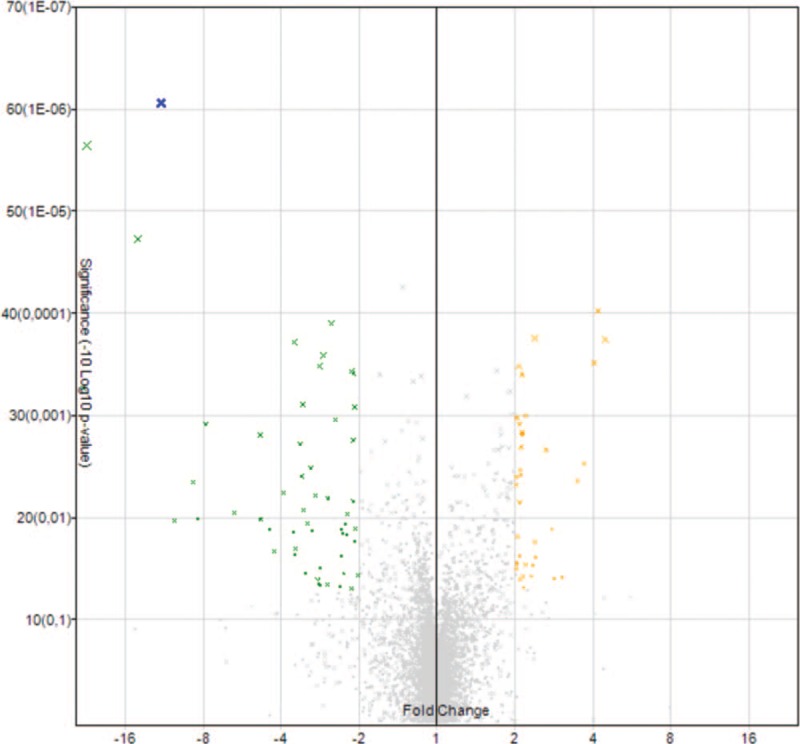
Volcano Plot. Deregulated miRNA in microarray analysis. The amount of microRNAs with statistically significant differences of expression can be appreciated in this graph. Those with a significantly higher expression are yellow highlighted and with a significantly lower expression green highlighted. *Fold change* is specified on the x-axis.

Eighty miRNAs exhibited statistically significant different expression levels in OSCC samples compared with healthy controls (Fig. 1); 35 and 45 miRNAs were upregulated and downregulated, respectively. In Table 2, deregulated miRNAs are summarized.

**Table 2:**
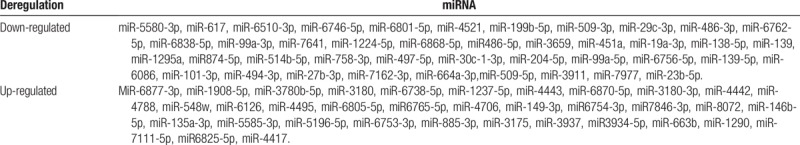
Up regulated and down regulated miRNAs in microarray analysis.

### 3.3 miRNA expression analysis by RT-qPCR

Following the screening criteria of a ±2.5-fold change and *P* values of <.01, and after in silico analysis of possible targets, four deregulated miRNAs were chosen for subsequent PCR analysis (miR-617, miR-497-5p, miR-4417, and miR-6825-5p). Only miR-497-5p maintained its diminished expression in all cases compared with controls (*P* = .01), while miR-4417 expression increased (*P* = .01).

As a complementary validation method, we performed a correlation study (Spearman rho) between miRNAs and the different platforms used, which yielded rho values of .65 and .84 for miR-497-5p CC (*P* = .02) and miR-4417 CC (*P* < .01), respectively.

### 3.4 Clinical characteristics and miRNA expression association

No significant association between patient sex and miR-497-5p or miR-4417 expression was found by the PCR analysis (*P* = .20 for miR-497-5p, *P* = .71 for miR-4417) or microarray analysis (*P* = .29 for miR-497-5p, *P* = .11 for miR-4417).

We detected significant differences between miR-4417 expression and nodular affectation (N1, 3.95; SD = .10 vs N0, 1.13; SD = 1.55; *P* = .03). We also found significantly diminished expression of miR-497-5p in patients who needed radiotherapy (*P* = .05).

The expression level of neither miRNA showed significant differences according to tumor localization (miR-497-5p, *P* = .47 for PCR and *P* = .11 for microarray; miR-4417, *P* = .21 for PCR and *P* = .16 for microarray). Expression distribution of both miRNAs was the same for differentiation, tumor size, tumor stage, metastasis, chemotherapy, relapse, or survival categories (Table 3).

**Table 3:**
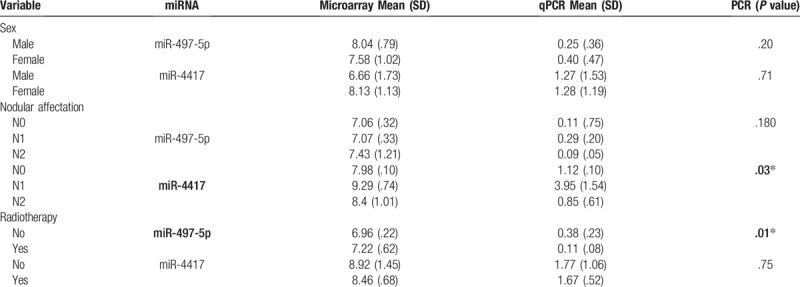
Association between miRNA expression and clinical parameters.

### 3.5 Survival analysis

The mean follow-up time for the patients was 52.71 months (SD = 71.13), with a range from 7.69 to 243.97 months. The average time of patient survival was 30.24 months (SD = 19.66), with a range from 11.51 to 73.46 months. To study the relationship between miRNA expression and survival, confirmed by PCR, we determined as a cut-off the mean expression level of both miRNAs. The mean expression values for miR-497-5p and miR-4417 were 0.149 and 1.71, respectively.

Cox regression analysis confirmed that patients with miR-497-5p expression levels below the mean value lived for an estimated 24.31 months (IC: 13.93–34.69) compared to those with higher expression levels (40.13 months; IC: 7.38–72.88), although these differences were not statistically significant (*P* = .53). Patients with miR-4417 expression levels above the mean value had a shorter survival (7.48 months; IC: 2.10–12.8) compared to patients with expression levels less than the mean value (12.86 months; IC: 3.35–22.38). However, these differences were not statistically significant either (*P* = .44).

### 3.6 KEGG pathway enrichment analysis

**Figure 2:**
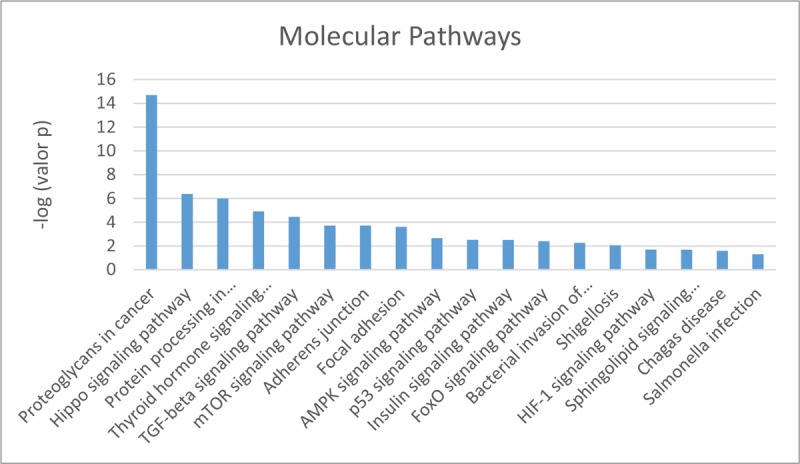
Molecular pathways in which deregulated miRNAs may be involved. Diana Tools results.

Among the 80 miRNAs that were found to be deregulated in the microarray analysis, we selected those whose *P* values were <.01 and searched them in Diana Tools. Only 6 of the screened miRNAs had known targets. MiRPath 3.0 showed 18 molecular pathways wherein those 6 miRNAs could be implicated. Figure 2 summarizes those molecular pathways with the significance levels. Other important molecular pathways studied in cancer previously also can be observed.

**Figure 3:**
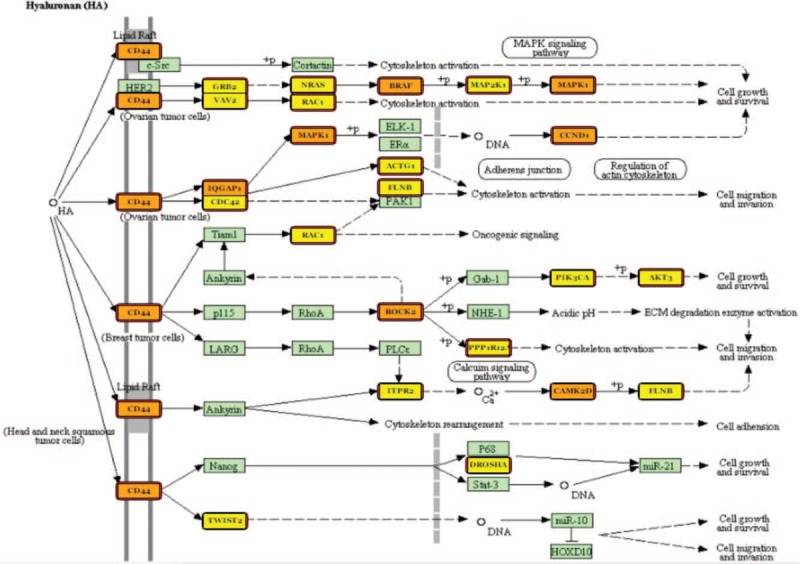
Hyaluronan (HA) proteoglycans molecular pathway and miRNAs deregulated in our study. Proteins target by one miRNA are coloured in yellow, and those targets by more than 1 miRNA are coloured in orange.

**Figure 4:**
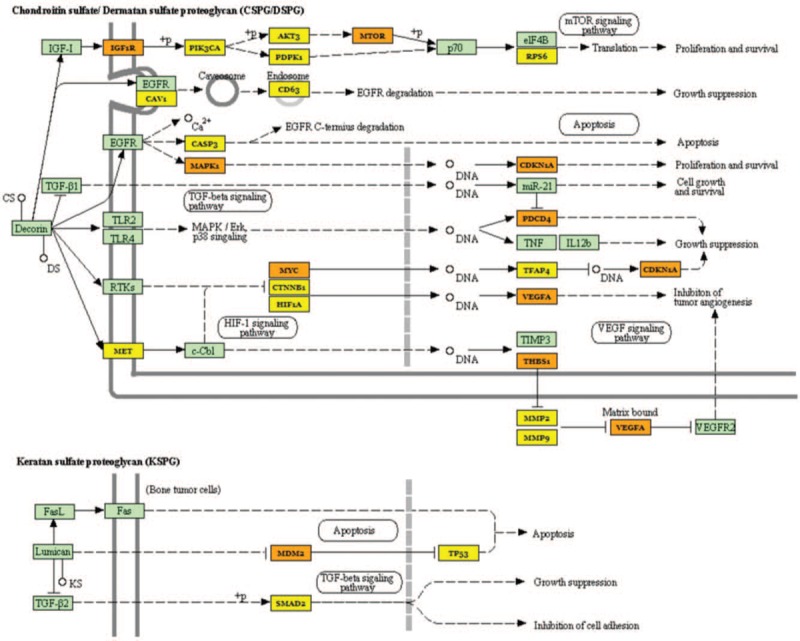
Chondroitin sulfate /Dermatan Sulfate (CSPG/DSPG) and Keratan Sulfate (KSPG) proteoglycans molecular pathway and miRNAs deregulated in our study. Proteins target by 1 miRNA are coloured in yellow, and those targets by more than 1 miRNA are coloured in orange.

**Figure 5:**
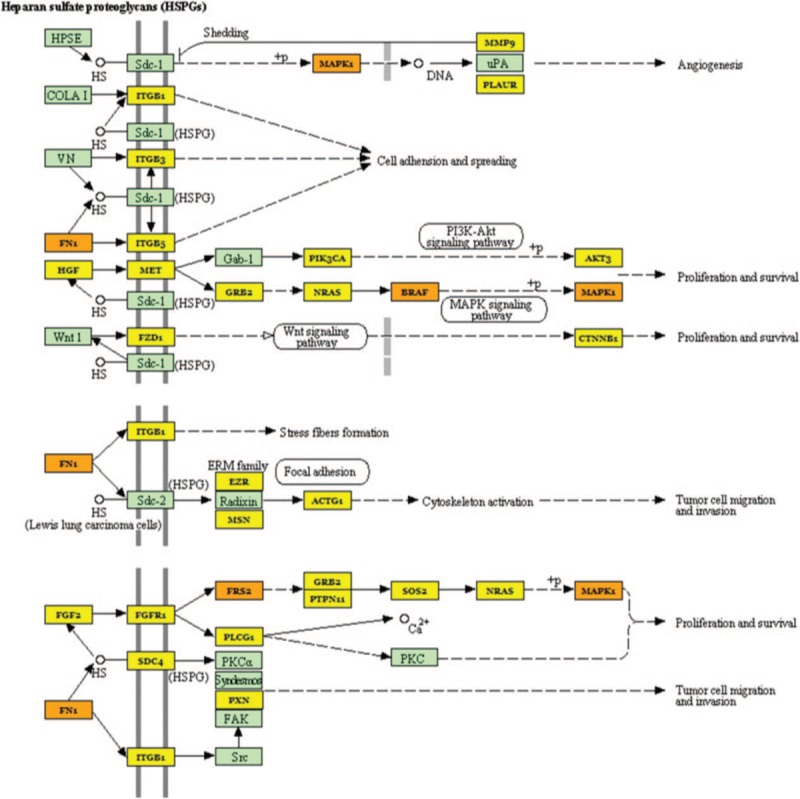
Heparan sulfate (HSPGs) Proteoglycans molecular pathway and miRNAs deregulated in our study. Proteins target by 1 miRNA are coloured in yellow, and those targets by more than 1 miRNA are coloured in orange.

We normalized the *P* values and confirmed that the most statistically significant was ‘proteoglycans in cancer’ (Figs. 3–5).

## 4 Discussion

Our results revealed 80 deregulated miRNAs in OSCC patients; four of them were validated by RT-qPCR, and only miR-497-5p and miR-4417 maintained their expression levels.

Our results of differential expression agree with some miRNA results of other profiling studies, but corresponded better with those of Setién-Olarra et al, who used a population similar to the one we used herein. They determined 92 deregulated miRNAs in OSCC patients, and 10 of them coincided with ours.^[13]^

In the past few decades, several studies have associated miRNA expression and clinical parameters in OSCC with recurrence, metastasis, and survival. However, to apply those results to clinical practice, more studies are needed. Lin et al carried out a meta-analysis to evaluate the potential of miRNAs as biomarkers to detect OSCC. They selected eight profiling studies and found different and contradictory results. They ascribed the heterogeneity among studies to differences in study design, sample size, sample type, and origin or population.^[10]^ Other recent meta-analyses of OSCC and miRNAs have been conducted by Zahra et al, Zeljic et al, and Troiano et al, which showed some correspondence and some differences in results.^[5,9,14]^ Authors conclude that a possible bias in meta-analysis is the origin of the samples. Most profiling studies of OSCC and miRNAs have been carried out in Asia. The meta-analysis by Troiano et al was the only one to include a study in Europe (Denmark).

OSCC prognosis can differ among ethnic groups, as carcinogenic patterns are different, for example, the mutation rates of *TP53* are significantly lower in Asian patients than in Caucasian patients.^[9]^

The need to perform more profiling studies of miRNA in OSCC in different parts of the world is justified by the lack of evidence to facilitate expression patterns on the basis of which clinicians can use miRNAs as biomarkers or as possible OSCC therapies.

miRNAs are already in the clinical trial phase for treating other cancers as MRX34 for hepatic carcinoma,^[15]^ but owing to variations in tobacco consumption habits, environment, and microbiome influences in miRNA expression, we still do not have enough evidence to apply the same findings to OSCC.^[16]^ The statistical analysis in our study revealed a relationship between low miR-497-5p expression (lower than the mean level) and radiotherapy requirement. Moreover, statistically significant over-expression of miR-4417 was found in patients with nodular affectation. These results suggest that the under-expression of miR-497-5p might be associated with more aggressive tumors that need more than only resection. One of the objectives of this study was to analyze the survival in relation to the expression of the studied miRNAs. However, analyses by Cox regression do not reflect statistically significant differences in survival. It is likely that the expression of these genes will not serve as prognostic biomarkers to determine survival; however, an increase in sample size, as we intend to develop, could reflect more clarifying results.

In the designing of our study, the mirVana miRNA isolation kit was chosen as the RNA isolation method because it permits the analysis of all RNAs of a sample, including those with <200 nucleotides. Although other methods exist, the mirVana miRNA isolation kit remains the most used.^[17]^ Furthermore, RNU6B was also used as an internal control for RT-qPCR as it has already been applied in other studies.^[18,19]^ Keratinized gingiva tissue was used as the control sample, because sometimes OSCC can also proceed from the gingiva, is exposed to risk factors in a manner similar to the other parts of the oral cavity, and taking it during the extraction of the third molar is a less invasive manner to obtain control tissue from healthy patients. In future studies, it would be better to use healthy samples of the same type of tissue (buccal mucosa, soft palate, retromolar region, tongue, and floor of the mouth), thus matching the tissue origin of the OSCC samples. Also, sample size could be greater, our preliminary results are very useful to analyze miR-497-5p and miR-4417 in future studies in a prospective way.

Diana Tools revealed many miR-497-5p targets related to OSCC. Characteristically, a single miRNA can act on different targets, and the same gene can be targeted by different miRNAs, this fact makes working with miRNAs difficult. CD44 is a surface glycoprotein implicated in interaction, proliferation, differentiation, cell migration, and angiogenesis. The over-expression of CD44 in saliva has been proposed as an OSCC biomarker, and CD44 concentration is reconsidered a likely risk factor.^[10,20]^ Insulin growth factor 1 receptor (*IGF1R*), another miR-497-5p target, is a transmembrane receptor activated by IGF1 and IGF2, and its over-expression has also been associated with OSCC.^[7,20]^ Other miR-497-5p targets are the *MYC* oncogene^[21]^ and *MMP9*,^[22]^ the high expression of which has long been studied in oral cancer.^[23]^ However, profiling studies from Asian patients have found miR-497-5p expression in OSCC patients as being upregulated.^[24,25]^ Our next step would be to measure CD44, MMP9 and MYC expression changes Western Blot after miR-497-5p inoculation.

Most deregulated miRNAs in this study have been identified only recently; therefore, there is still not enough evidence about their targets or the effects that can produce their altered expression in tissues. Among the 18 screened miRNAs with more significant values (*P* ≤ .001), only 6, all of them downregulated, had known targets. In silico enrichment analysis revealed different molecular pathways, like proteoglycans in cancer; protein processing in the endoplasmic reticulum; adherens junctions; focal adhesion; and the Hippo, thyroid hormone, TGF-beta, mTOR, AMPK, p53, insulin, FoxO, HIF-1, and sphingolipid signaling pathways. Most of them are known pathways implicated in tumor development.^[26]^ This fact confirms the association between under-expressed miRNAs and oncogenes.

Proteoglycans are the main component in connective tissue that can be linked to the plasma membrane or extracellular matrix, and permit motility and cellular migration and filter the substances that can be transported inside the cell. Proteoglycans in the tumor microenvironment have been demonstrated to participate in proliferation, angiogenesis, or metastasis. For example, Perlecan, a heparan sulfate or sulphatase 2, is over-expressed in cancerous and precancerous oral lesions.^[27,28]^

Our study demonstrates that the expression of miRNAs in OSCC can be used as a prognostic biomarker, but the differences in expression levels among different populations is a field of research that requires further investigation. Possibly, the use of big data is necessary to be able to take advantage of these small RNAs as a tool for OSCC diagnosis and prognosis, and this study contributes to make this analysis possible in the future.

## Acknowledgments

We wish to thank our colleagues from the Maxillofacial Surgery Unit, the Pathological Anatomy Unit and the biobank of SERGAS in the Santiago de Compostela Teaching Hospital.

## Author contributions

**Conceptualization:** Mario Pérez-Sayáns, Abel Garcia Garcia.

**Data curation:** Alejando Ismael Lorenzo Pouso.

**Formal analysis:** Jose Manuel Suarez-Peñaranda.

**Investigation:** Maria Elena Padin-Iruegas.

**Methodology:** Andres Blanco Carrion.

**Supervision:** Abel Garcia Garcia.

**Validation:** Cintia Micaela Chamorro Petronacci.

**Writing – original draft:** Cintia Micaela Chamorro Petronacci.

**Writing – review & editing:** Mario Pérez-Sayáns.

Cintia Micaela Chamorro Petronacci orcid: 0000-0002-5637-9908.
